# ADAPTING A DISASTER PREPAREDNESS PROGRAM USING AN INDIGENOUS IMPLEMENTATION FRAMEWORK

**DOI:** 10.1093/geroni/igae098.1468

**Published:** 2024-12-31

**Authors:** Lena Thompson, Jordan Lewis, Sato Ashida

**Affiliations:** University of Minnesota Duluth, Duluth, Minnesota, United States; University of Alaska Fairbanks, College of Indigenous Studies, Fairbanks, Alaska, United States; University of Iowa, Iowa City, Iowa, United States

## Abstract

Implementation frameworks can help increase the relevance and sustainability of intervention research with Indigenous populations. The He Pikinga Waiora (HPW) framework centers around Māori self-determination and has four elements: community engagement, cultural centeredness, integrated knowledge translation, and systems thinking. This study applied the HPW framework to inform adaptation of a disaster preparedness program for older adults with a Midwestern tribe in the United States. Tribal organization leaders, including a member from the Tribal Government, Senior Center, Emergency Preparedness Department, Police Department, and one older adult from the community, read through the program with the researcher while considering the four elements of HPW throughout the process. Five experts in Indigenous aging research and practice listened to the recordings of these sessions and evaluated the researcher’s fidelity to the framework and the extent to which the HPW framework fit the community context and project. Additionally, research assistants conducted qualitative interviews with Tribal organizational leaders about the fit and relevancy of the framework. Data from expert evaluators and interviews with tribal organizational leaders and the community member suggest that the HPW framework was a good fit for the community and program. Both groups made recommendations to improve the framework, including integrating sustainability into the model and making language simpler. The HPW framework appears to facilitate healthy partnerships and provide guidance to adapt, implement, and eventually disseminate a tailored intervention to a Midwestern Tribal community. This study demonstrated beginning processes of conducting cultural adaptations using an implementation model created and tested with Indigenous populations.

